# Preparation of Nanofibrous PVDF Membrane by Solution Blow Spinning for Mechanical Energy Harvesting

**DOI:** 10.3390/nano9081090

**Published:** 2019-07-30

**Authors:** Rui-Qiang Liu, Xiao-Xiong Wang, Jie Fu, Qian-Qian Zhang, Wei-Zhi Song, Yuan Xu, You-Qiang Chen, Seeram Ramakrishna, Yun-Ze Long

**Affiliations:** 1 Collaborative Innovation Center for Nanomaterials & Devices, College of Physics, Qingdao University, Qingdao 266071, China; 2 State Key Laboratory of Bio-Fibers and Eco-Textiles, Qingdao University, Qingdao 266071, China; 3 Center for Nanofibers & Nanotechnology, Faculty of Engineering, National University of Singapore, Singapore 119077, Singapore

**Keywords:** solution blow spinning, micro-energy harvesting, nanogenerators

## Abstract

Self-powered nanogenerators composed of poly(vinylidene fluoride) (PVDF) have received much attention. Solution blow spinning (SBS) is a neoteric process for preparing nanofiber mats with high efficiency and safely, and SBS is a mature fiber-forming technology that offers many advantages over conventional electrospinning methods. Herein, we adopted the SBS method to prepare independent PVDF nanofiber membranes (NFMs), and successfully employed them as nanogenerators. Finally, we tested the change in the output current caused by mechanical compression and stretching, and studied its durability and robustness by charging the capacitor, which can drive tiny electronic devices. The results show that the PVDF nanogenerators by using this SBS equipment can not only be used in wearable electronic textiles, but are also suitable for potential applications in micro-energy harvesting equipment.

## 1. Introduction

Self-powered systems are systems that collect energy to operate on their own, and can run continuously without an external power supply; they are widely used in data transmission, detection, data processing and sensing [1,2,3,4]. Nanogenerators (NG) are systems that collect tiny amounts of mechanical energy to convert into electrical energy; they are used in artificial skins [5,6,7], self-powered wearable electronics [8,9,10,11,12], medical science, and security [13,14,15,16,17].

Polyvinylidene fluoride (PVDF) has many excellent properties, including ferroelectricity, piezoelectricity, pyroelectricity and dielectric properties [18]. PVDF is a free-radically polymerized polymer containing four different crystal structures defined as α, β, γ and δ phases, respectively. The formation of crystalline forms is related to three different chain structures [19,20]. Generally, the α phase in the crystal phase composition of PVDF occupies a large proportion. However, the α phase is conformed by the (TGTG) molecular chain, resulting in the α phase exhibiting non-ferroelectric characteristics. Among the three polarization phases, the β phase has an all-trans (TTTT) zigzag chain conformation, in which the dipole is perpendicular to the chain axis, so it exhibits the largest dipole moment, resulting in relatively high spontaneous polarization, Therefore, the β phase exhibits a strong piezoelectric response. It shows piezoelectricity, pyroelectricity and ferroelectricity, giving PVDF the potential for a wide range of device applications [20,21]. It has been proved that large strain stretching or high electric field polarization can promote α→β phase conversion [22,23,24,25]. 

**Figure 1:**
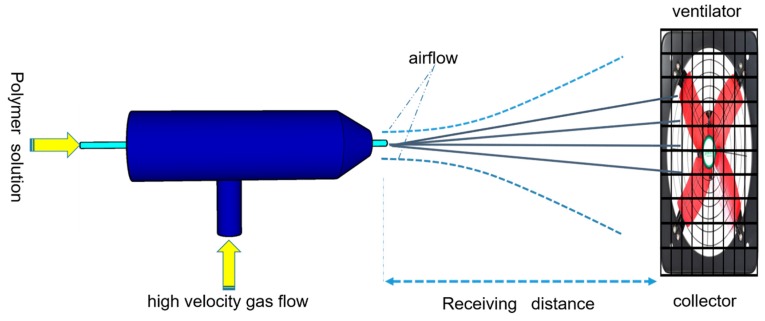
The preparation process of PVDF nonwoven membrane via solution blow spinning.

Solution blow spinning (SBS) technology is a new technology that has emerged in recent years; it is a new nanofiber preparation method based on the principle of high speed airflow drawing, which has the advantages of having a simple process, low energy consumption and high production efficiency compared with the electrostatic spinning method, and it has been widely applied in the fields of filtration materials, protective materials, medical dressings and proton exchange membranes [26,27,28,29]. The SBS device, as shown in Figure 1, works by supplying the spinning solution to the nozzle at a certain rate, and then the solution is squeezed out of the nozzle to form a fine flow of the solution. Under the action of the high-speed airflow field, the fine flow is gradually stretched and becomes thinner, accompanied by the evaporation of the solvent, and finally the curing formation of nanofibers is collected on a specific collection device. 

In this work, we report the use of high-pressure airflow to provide a large strain force to stretch the PVDF precursor in order to increase the amount of β phase in the PVDF nanofiber membrane. In addition, intact PVDF nanofiber membrane was prepared using the solution blow spinning (SBS) method. In addition, we report self-powered non-woven nanogenerators composed of solution-blow-spinning PVDF nanofibrous membrane and electrospinning PVA/PEDOT:PSS conductive nanocomposite fiber membrane (CNCFM). The electrospinning PVA/PEDOT:PSS CNCFM is used as an electrode to verify that flexible conductive non-woven fabrics can be used as electrodes, proving that the device can be used in wearable electronic textiles. In addition, we experiment with the piezoelectric fabrication of nanogenerators caused by mechanical squeeze and bending and the fabrication changes caused by different squeeze/bending frequencies. The mechanical durability, robustness and practicability of the capacitors are investigated by charging them. These results indicate that our apparatus has a large range of applications in self-powered nonwovens and micro-scale energy harvesting devices.

## 2. Experimental

### 2.1. Materials

poly(vinylidene fluoride) powder (Mw~550,000, Shanghai, China), Polyvinyl Alcohol (PVA, M_W_~67,000, Aladdin, Shanghai, China) and poly(3,4-ethylenedioxythiophene):poly(styrene sulfonate) (PEDOT:PSS) dispersion liquid (1.05 wt% dispersion in water, Shanghai, China) were used in this work. *N*,*N*-dimethylformamide (DMF) and ethanol (Sinopharm Chemical Reagent, Shanghai, China), Acetone (Laiyang Fine Chemical Factory, Laiyang, China). copper foil tape (double-sided conductive, Dongguan, China).

### 2.2. Fabrication of PVDF Nonwoven Membrane

**Figure 2:**
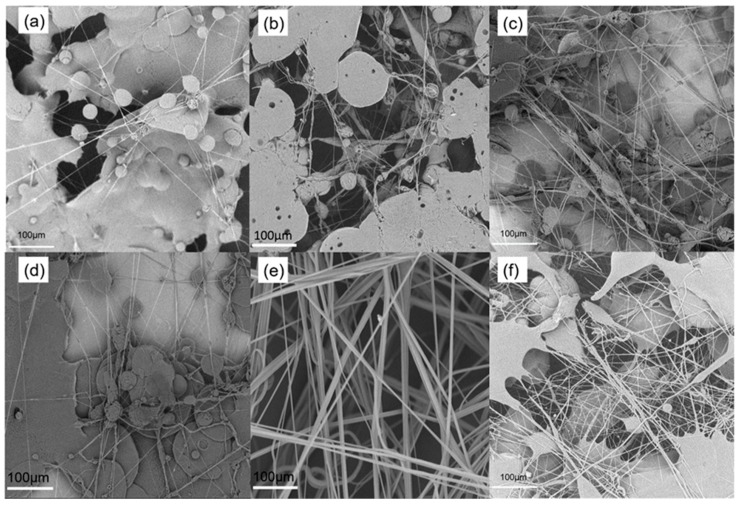
SEM for different concentrations and solvents of PVDF: (**a**) DMF/15 wt%, (**b**) DMF/20 wt%, (**c**) DMF/25 wt%, (**d**) DMF-acetone/15 wt%, (**e**) DMF-acetone/20 wt%, and (**f**) DMF-acetone/25 wt%.

To explore the preparation of PVDF nanofibrous membrane by SBS, we selected three concentrations of PVDF solution: 15 wt%, 20 wt%, and 25 wt%. The solvents were pure DMF and DMF-acetone (1:2 *w*/*w*), and the other experimental conditions were the same. Figure 2 shows a fiber morphology diagram obtained from six precursor solutions. Finally, according to the experimental results, the optimum shape of the precursor is selected to PVDF nanofiber membranes. 

The SBS technology is based on air flow to drive the solution to stretch; therefore, as shown in Figure 2a–c, the precursor solution is volatilized slowly with pure DMF as the solvent, and the fiber cannot solidify. Therefore, when using a faster evaporation time for the acetone solvent and DMF solvent, the precursor solution was more volatile and solidified. In the case of the acetone-DMF mixed solvent, the precursor concentration was not sufficiently low and the fiber could not be formed, as shown in Figure 2d. The morphology and structure of the fiber in Figure 2e is optimal. The precursor has a high solubility, the airflow traction was insufficient, and the fibers could not be formed, as shown in Figure 2f. Therefore, we selected the corresponding acetone–DMF/20 wt% precursor ratio for PVDF nanofiber membranes.

### 2.3. Preparation of Spinning Solution

Firstly, the PVDF powder was dissolved in an acetone-DMF solvent mixture (1/2 *w*/*w*), and then the mixture was stirred with a magnetic stir bar at 45 °C for 5 h to obtain a PVDF precursor (20 wt%). Secondly, preparation of a precursor solution of a conductive nanofiber membrane by mixing PEDOT: PSS conductive liquid in a PVA precursor. The 5 g PVA powder is added to 35 g water and stirred to form a transparent PVA solution. The 10 g PEDOT:PSS is then added to the solution above. After stirring for 3 h, the solution is mixed evenly.

### 2.4. Fabrication of the Nanogenerators

Firstly, the precursor PVDF nanofiber membrane was prepared using a self-assembled SBS device. The PVDF precursor solution is loaded with a syringe with a coaxial nozzle. The solution stream is squeezed from the inner nozzle using a syringe pump, and the high velocity gas stream is rapidly drawn and pulled. The exhaust fan covered by the shielding window is used as the collector of the nanofiber, and the exhaust fan speed is 3100 rpm. The injection rate is 3 mL h^−1^, the tip-to-collector distance was fixed at 50 cm, and the gas pressure was fixed at 0.4 MPa. After a period of time, the gained PVDF nanofiber membrane was peeled off carefully from the screen window.

Secondly, a syringe with a PVA/PEDOT:PSS solution was placed in a syringe pump (LSP01-1A, Baoding, China) with a flow rate of 1 mL h^−1^. The aluminum foil electrode was covered with a PVDF nanofiber film prepared in advance as a current collector for the electrostatic spinning PVA conductive solution, the collection distance was 20 cm, and a voltage of 12 kV was applied in between the needle and the collector. Thus, a device with a three-layer structure comprising PVA/PEDOT—a PSS conductive nanocomposite fiber membrane (CNCFM), PVDF nanofiber membrane and aluminum foil—was obtained.

**Figure 3:**
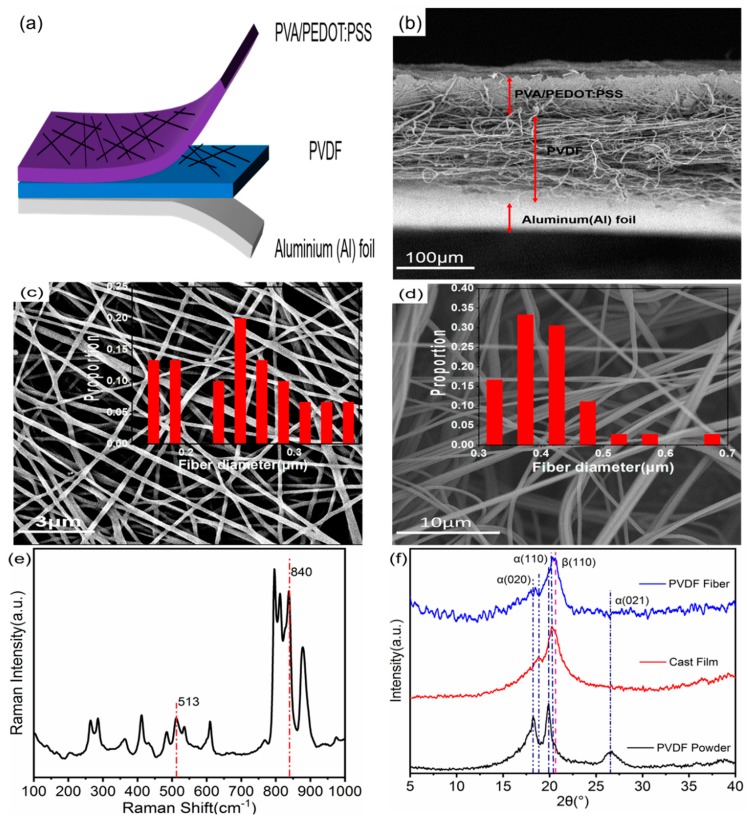
(**a**) Schematic diagram of nanogenerator structure. (**b**) Cross-section SEM image of the nanogenerator structure. (**c**) SEM images and bar charts (insets) of the fiber size distributions of the PVA/PEDOT:PSS CNCFM and (**d**) SBS pure PVDF nanofiber membrane. (**e**) Raman spectra of the SBS pure PVDF nanofiber membrane. (**f**) XRD patterns of PVDF powder, PVDF cast film and SBS nanofibers.

Finally, a rectangular sample measuring 4 cm × 4 cm was cut from the three-layer structure device. Aluminum foil is used as the substrate, PVA/PEDOT:PSS CNCFM covering the PVDF nanofiber membrane is used as the upper electrode, and copper foil tape is connected to the upper and lower electrodes. To prevent the device structure from being contaminated, an ordinary adhesive tape was adopted to package the entire NG. Meanwhile, this sensor was squeezed with an appropriate pressure to eliminate the gaps between the membranes, aiming to avoid the triboelectric effect. The structure of the nanogenerator equipment is shown in Figure 3a. We measured the capacitance of NG (Agilent 4268B, Agilent Technologies, Beijing, China) to be 142.5 pF at 1000 Hz.

### 2.5. Characterization

Characterization of the morphology and microstructure of the sample using scanning electron microscopy (SEM, JSM-6390, JEOL, Tokyo, Japan). PVDF and CNCFM fiber diameters were calculated using Nano Measurer 1.2.5 software(Free sharing software, Shanghai, China). The crystal structure of the PVDF nanofiber membrane was analyzed on a diffractometer (Rigaku, SmartLab, Tokyo, Japan) and Raman spectroscopy were collected by the Laser Raman Spectroscopy (Labram HR 800, Jobin-Yvon Horiba, Kyoto, Japan). X-ray diffraction (XRD) and Raman spectra were obtained. In addition, the output voltage is monitored by a digital multimeter (Rigol DM 3058, Beijing, China). The picoammeter (Keithley 6487, Everett, WA, USA) monitors the change in output current and monitors the real-time charging voltage of the capacitor through a digital multimeter (Rigol DM 3058, Beijing, China). Self-made pressure devices and curved straightening devices were capable of producing periodic changes [30].

## 3. Results and Discussion

### 3.1. Basic Characterization of the Nanogenerator

Figure 3a is a schematic diagram of the structure of a self-powered system that is based on the SBS nonwoven PVDF nanofibrous membrane. The system is composed of three layers (the cross-sectional structure of the three-layer equipment is shown in Figure 3b), two of the layers are PVA/PEDOT:PSS CNCFM prepared by electrospinning and PVDF nanofiber membrane obtained by SBS. The other layer is the aluminum foil used as the substrate. The PVA/PEDOT:PSS CNCFM acts as the upper electrode. PVA/PEDOT:PSS CNCFM has a thickness of about 50 μm and an average fiber diameter of about 250 nm, as shown in Figure 3c. In this work, the use of PVA/PEDOT:PSS CNCFM as an electrode, which not only doesn’t affect the PVDF nanofibers, but can also be easily attached to the PVDF nanofiber membrane. The flexible features it shows can be applied to wearable electronic textiles.

The SBS PVDF nanofiber membrane is the core part of the nanogenerator, with a thickness of about 150 μm. As can be seen from Figure 3d, the average fiber diameter of PVDF nanofiber membranes is about 400 nm. The PVDF nanofiber membrane β phase has a great influence on the piezoelectric properties. Therefore, the crystallization behavior of PVDF nanofiber membranes was characterized by XRD and Raman spectroscopy, and a more comprehensive phase characterization was obtained. For comparison, a layer of PVDF solution (20 wt%) was spread on an aluminum foil sheet at room temperature to obtain a PVDF flow film. The XRD pattern of PVDF powder and PVDF flow film is shown in Figure 3f. It can be obtained from the curve in the figure that the PVDF powders exhibited significant peaks at 2θ = 18.30°, 19.90° and 26.56°, which correspond to the reflection peaks of the α phase (020), (110) and (021), respectively [31,32]. The PVDF flow film showed distinct peaks at 2θ = 18.8° and 20.2°, and the α phase (020) and (110) reflection peaks correspond to one another. In the curve of SBS PVDF nanofiber membranes, these peaks are decreased sharply, while at the same time, a new peak appears at 2θ = 20.26°, corresponding to the (110) reflection formed by the β phase [33,34,35], consistent with the results shown by the Raman patterns [36], as shown in Figure 3e. This shows that the preparation of pure PVDF nanofibers with SBS is beneficial to the formation of β phase, this discovery is also reflected in previous reports [23,37,38]. This is due to the high viscosity of the precursor solution during SBS; the rapid traction and stretching of the airflow results in high enough stress, thus destroying the crystallization sequence. When the stretching stops, the solution evaporates rapidly, and the crystal is reorganized into the most stable crystalline phase. The β phase in environments below 80 °C is the most stable phase, so in the working environment at room temperature, the β phase crystallization rate was higher than the α phase. Therefore, the α phase in SBS pure PVDF is transformed into β phase, which contributes to the constitution of the structure of the β phase in PVDF and improves the piezoelectric properties of the PVDF nanofiber membrane.

### 3.2. Piezoelectric Outputs of the Nanogenerator

**Figure 4:**
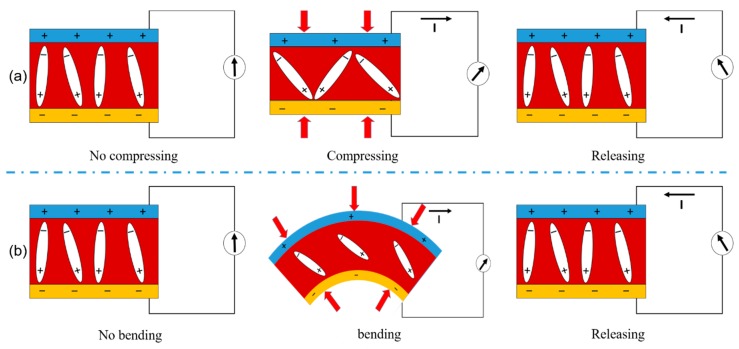
Schematic diagrams of the piezoelectricity of the solution-blow-spinning PVDF nanofibers. (**a**) Principles of piezoelectric output in compressing/releasing mode of PVDF fibers. (**b**) Principles of piezoelectric output in bending/releasing mode of PVDF nanofiber.

It has previously been reported that PVDF can exhibit piezoelectric properties without participating in polarization [39,40,41,42]. It can be seen that polarization can also exist without the poling process, because the electrode strength cannot be strictly offset due to the disordered arrangement of the electric dipole moments. Of course, the polarization strength in different samples is also different, and is small relative to the polarized material. To more clearly represent the experimental results, the energy conversion mechanism of the nanogenerator is shown in Figure 4. At the beginning, there is no piezoelectric output when the sensor is not impacted or vibrating, because the electric dipoles in PVDF randomly oscillate on their respective alignment axes, their total average spontaneous polarization remains in a macro equilibrium state [43,44]. When the nanogenerator is subjected to vertical mechanical shock, the dipole is pressured to form a potential difference between two electrodes and to produce an electron flow between two electrodes, i.e., the first signal observed. Conversely, when the impact disappears, the deformation of the nanogenerator restored, the spontaneous polarization recovered to the original state, and the second signal can be detected. The release process is shown in Figure 4a. Similarly, in the process of mechanical bending, nanogenerator can also produce piezoelectric current output. The energy conversion mechanism of nanogenerator during mechanical bending is shown in Figure 4b. Mechanical deformation results in lateral strain on NG and decreases polarization along the direction of polarization. To balance the induced charge, an electron transfer (positive current) from the bottom electrode to the top is generated. As the device restores the equilibrium state from the bending state, the decreasing mechanical strain recovering electrode, lead to the reverse motion of the electron [45].

**Figure 5:**
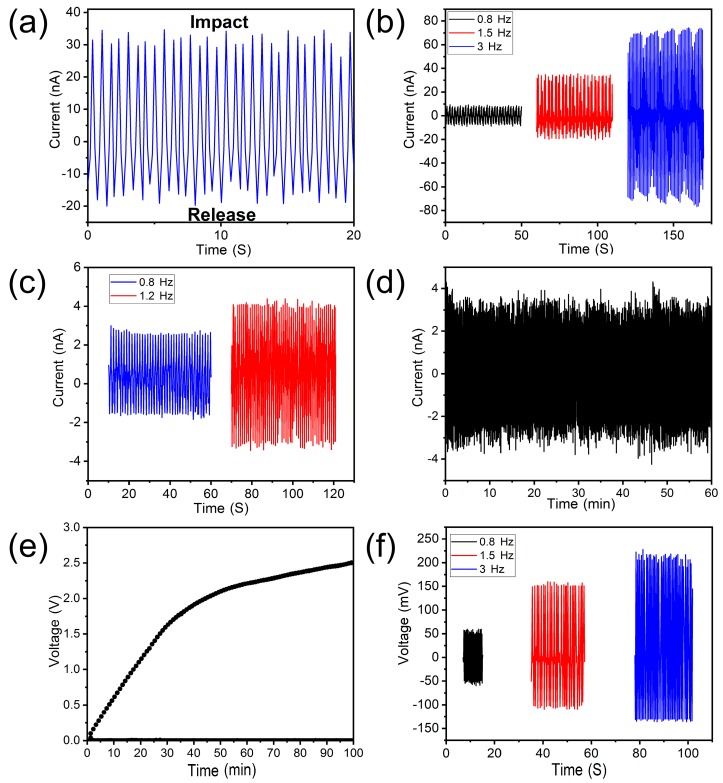
(**a**) Output current of the SBS PVDF non-woven under 1.5 Hz repeated compressive impacts. (**b**) Influence of compression frequency on the output current of the nanogenerator. (**c**) Influence of mechanical bending on the output current of the nanogenerator. (**d**) The durability test results of the nanogenerator subjected to a bending frequency of 1.2 Hz. (**e**) The voltage of the 47 μF capacitor versus charging time for the nanogenerator to be operated under 0.8 Hz bend frequency. (**f**) Influence of compression frequency on the output voltage of the nanogenerator.

In order to determine the piezoelectric export of nanogenerator devices, we used the repeated compressions of our homemade pressure devices and the release of nanogenerators. The nanogenerator was repetitively compressed and released by a self-assembled pressing apparatus at a frequency of 1.5 Hz. The output current is shown in Figure 5a as a typical piezoelectric signal. When the nanogenerator is pressed under a certain pressure, a pulsed current/voltage signal (denoted as the first signal) is generated. When the pressure is released, the opposite pulse signal (denoted as the second signal) is generated. In addition, we also studied the effect of the increase of external force frequency on the output current. Under conditions in which the collision produces pressure, the mass of the mass module does not change, the frequency increases, the speed of the mass module increases, and the impact force is different, the output voltage will change with the frequency. As shown in Figure 5b, at a frequency of 0.8 Hz, the average positive peak and the negative peak output current are about 9.5 nA and −8.6 nA, respectively. When the compression frequency is 1.5 Hz, the positive/negative current output reaches 30 nA and −20 nA, while the positive/negative peaks are 70 nA and −65 nA at an impact frequency of 3 Hz. Furthermore, we studied the influence of the compression frequency on the output voltage, and discovered that the trend was the same as that of the current, as shown in Figure 5f.

Furthermore, we studied the effect of bending frequency on the output current using a self-made mechanical bending device. The bending radius was 0.58 cm and the bending angle was 55°. As shown in Figure 5c, when the bending frequency of nanogenerator was 0.8 Hz, the average positive/negative peaks of nanogenerator were 3 nA and −1.6 nA, respectively. When the bending frequency was increased to 1.2 Hz, the corresponding peaks were 4 nA and −3 nA. These results indicate that an increase in the bending frequency leads to an increase in the exit current. As shown in Figure 5d, the nonwoven nanogenerator has mechanical durability, with a bending frequency cycle of 1.2 Hz for one hour, and its output current remains relatively constant with no significant attenuation of the magnitude of the current.

## 4. Application to Harvesting Energy and Drive Electronic Devices

**Figure 6:**
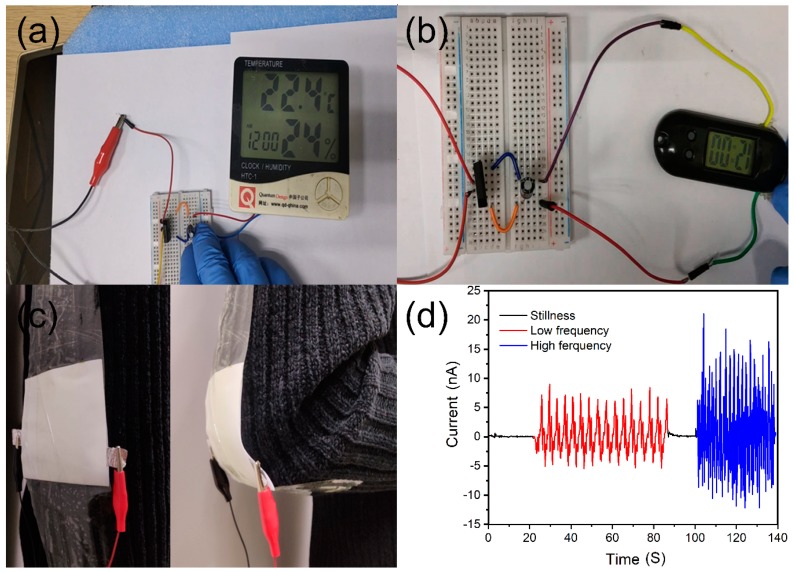
Electronic devices driven by NG, such as (**a**) temperature-humidity sensor, (**b**) electric watch. (**c**) Photograph of the device fixed to an elbow, and (**d**) the output currents generated by arm swinging at low frequency and high frequency.

The energy generated by this lightweight self-powered nonwoven NG can be stored in commercial capacitors. The energy generated by the nanogenerator can charge a 47 μF commercial capacitor. Micro-portable electronic devices can operate on charging voltages, hence playing an important role in the development of portable self-powered devices. Nanogenerators, as energy generation devices and capacitor combinations, can drive many electronic devices to work properly. As shown in Figure 6a,b, we used temperature-humidity and electronic watches for the purposes of demonstration. Figure S2 shows the output current and output power of NG with various external load resistances. The operational demonstration (Videos S1 and S2) can be found in the Supplemental information. Obviously, NG has a sensitive energy harvesting capability (as shown in Figure S3), so NG has a great advantage in powering portable microelectronic devices and can promote the rapid development of NG.

Additionally, this lightweight NG provides energy for carrying electronic devices and can be worn on the body. The movement of the human body drives the bending of human joints, such as the wrists, elbows, knees and cervical vertebrae, which can cause mechanical deformation of the NG, generate energy, and store it in commercial capacitors. We tested the current output produced in the swinging arms of the two frequencies using elbow bending as an example. The operational demonstration (Video S3) can be found in the Supplemental information. As shown in Figure 6c,d, the NG is taped to the elbow position, when the swing arm frequency is about 0.3 to 0.4 Hz, it corresponds to the swing arm frequency when walking, and the current output range is −4 nA to 6 nA. When the frequency of swinging arm is increased to a high frequency of about 1 to 1.1 Hz, which is equivalent to the frequency of swinging arms in a jogging motion, the current output range reaches −10 nA to 15 nA. Considering the wide application of the nanofiber membrane in textile fabrics and the potential application of SBS technology in large-scale preparation of nanofiber membrane, large-area self-generating textiles can be manufactured using SBS technology.

## 5. Conclusions

In summary, we successfully prepared PVDF nanofiber membranes by the solution blowing spinning process and prepared them into nanogenerators with potential applicability in large nanogenerators. The SBS PVDF nanofiber membrane is the working material, with PVA/PEDOT:PSS CNCFM and aluminum foil being the electrodes of the nanogenerator. The output current of the nanogenerator at different mechanical shock and bending frequencies was measured. Nanogenerators can extract energy from human motion. Electricity generated by nanogenerators can be used to charge capacitors and drive tiny electronic devices. All of these results demonstrated that this new type of high-efficiency SBS production of PVDF nonwoven membrane have potential applications in the large-scale preparation of micro-scale energy collection and wearable electronic products.

## Supplementary Materials

The following are available online at https://www.mdpi.com/2079-4991/9/8/1090/s1, Figure S1: 0.8 Hz frequency change voltage output, Figure S2: Output current and output power of NG with various external load resistances, Figure S3: NG’s sensing size for different weights, Video S1: Temperature-humidity sensor driven by NG, Video S2: Electric watch driven by NG, Video S3: The device fixed to an elbow.
