# Supplementary material for: Preparation of Nanofibrous PVDF Membrane by Solution Blow Spinning for Mechanical Energy Harvesting
Source: Nanomaterials (Basel). 2019 Jul 30;9(8):1090. doi: 10.3390/nano9081090 (PMC6723452; doi:10.3390/nano9081090)
Supplement: Supplementary file 1 [file nanomaterials-09-01090-s001.pdf]

nanomaterials

Article

Preparation of Nanofibrous PVDF Membrane by Solution Blow Spinning for Mechanical Energy Harvesting

Rui-Qiang Liu [1,†], Xiao-Xiong Wang [1,†], Jie Fu [2], Qian-Qian Zhang [1], Wei-Zhi Song [1], Yuan Xu [1], You-Qiang Chen [1], Seeram Ramakrishna [1,3] and Yun-Ze Long [1,*]

[1] Collaborative Innovation Center for Nanomaterials & Devices, College of Physics, Qingdao University, Qingdao 266071, China
[2] State Key Laboratory of Bio-Fibers and Eco-Textiles, Qingdao University, Qingdao 266071, China
[3] Center for Nanofibers & Nanotechnology, Faculty of Engineering, National University of Singapore, Singapore 119077, Singapore
* Correspondence:Correspondence: yunze.long@163.com or yunze.long@qdu.edu.cn; Tel.: +86-139-5329-0681; Fax: +86-532-8595-5977
† These authors contributed equally to this work.

Received: 9 June 2019; Accepted: 24 July 2019; Published: date

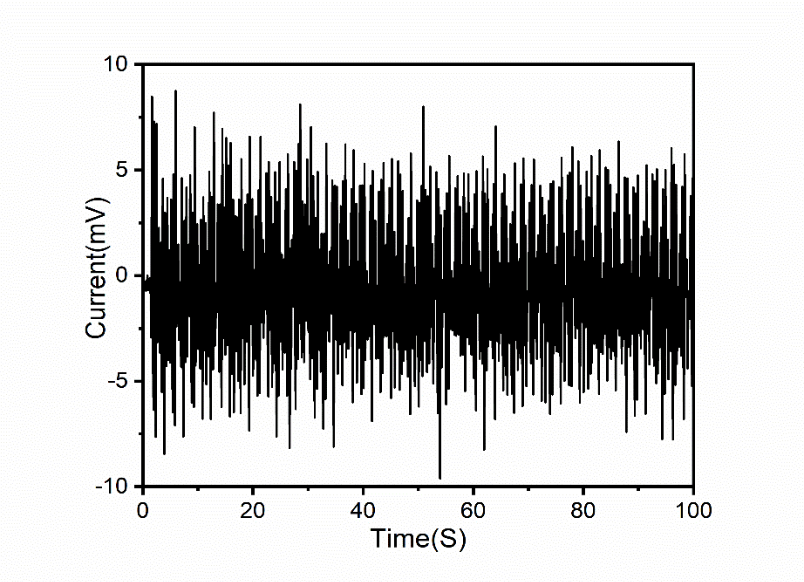

Figure S1. 0.8Hz frequency change voltage output (Rigol DM 3058).

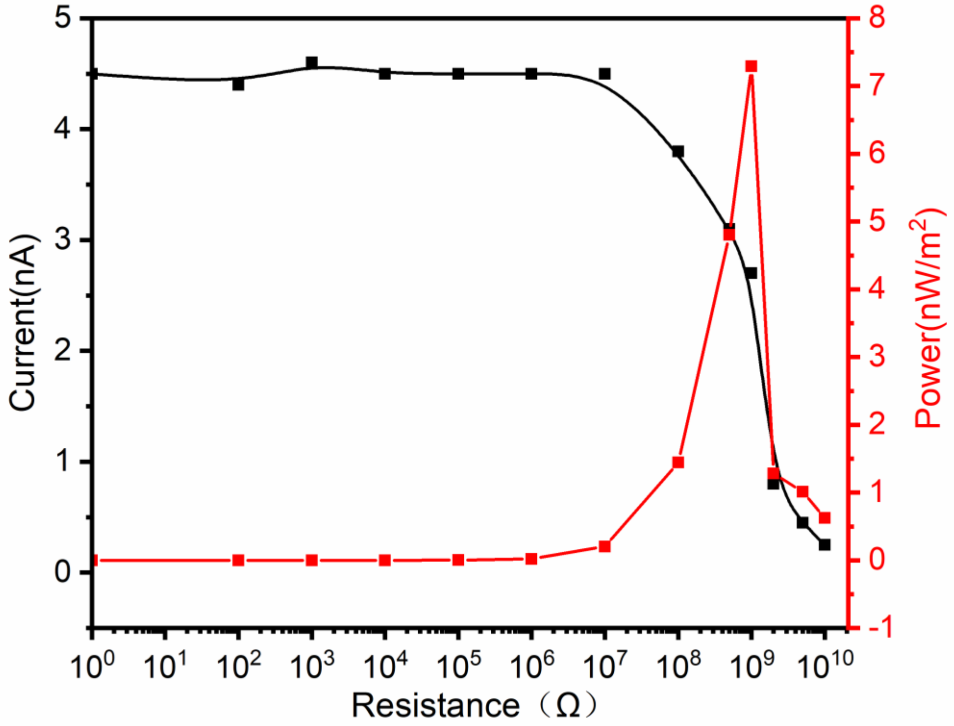

Figure S2. Output current and output power of NG with various external load resistances.

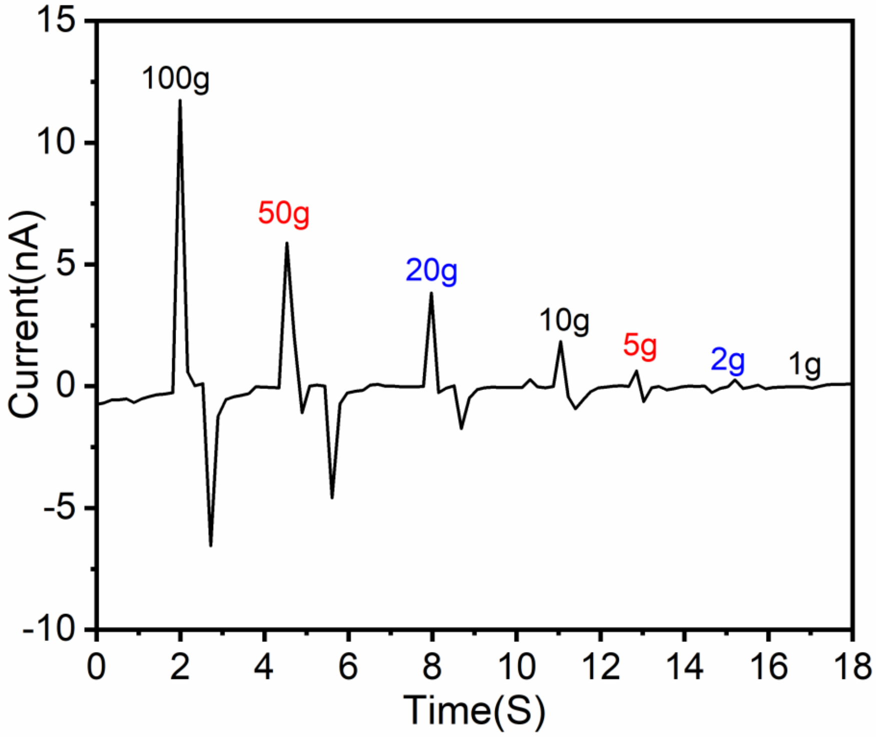

Figure S3. NG's sensing size for different weights.
